# Prognostic Value of Polycomb Proteins EZH2, BMI1 and SUZ12 and Histone Modification H3K27me3 in Colorectal Cancer

**DOI:** 10.1371/journal.pone.0108265

**Published:** 2014-09-22

**Authors:** Anne Benard, Inès J. Goossens-Beumer, Anneke Q. van Hoesel, Hamed Horati, Hein Putter, Eliane C. M. Zeestraten, Cornelis J. H. van de Velde, Peter J. K. Kuppen

**Affiliations:** 1 Department of Surgery, Leiden University Medical Center, Leiden, The Netherlands; 2 Department of Medical Statistics, Leiden University Medical Center, Leiden, The Netherlands; University of Maryland School of Medicine, United States of America

## Abstract

Numerous changes in epigenetic mechanisms have been described in various types of tumors. In search for new biomarkers, we investigated the expression of Polycomb-group (PcG) proteins EZH2, BMI1 and SUZ12 and associated histone modification H3K27me3 in colorectal cancer. Nuclear expression of PcG proteins and histone modification H3K27me3 were immunohistochemically (IHC) stained on a tissue microarray (TMA), including 247 tumor tissues and 47 normal tissues, and scored using the semi-automated Ariol system. Tumor tissues showed higher expression of EZH2 (p = 0.05) and H3K27me3 (p<0.001) as compared to their normal counterparts. Combined marker trend analyses indicated that an increase in the number of markers showing high expression was associated with better prognosis. High expression of all four markers in the combined marker analyses was correlated with the best patient survival and the longest recurrence-free survival, with overall survival (p = 0.01, HR 0.42(0.21–0.84)), disease-free survival (p = 0.007, HR 0.23(0.08–0.67) and local recurrence-free survival (p = 0.02, HR 0.30(0.11–0.84)). In conclusion, we found that expression of PcG proteins and H3K27me3 showed prognostic value in our study cohort. Better stratification of patients was obtained by combining the expression data of the investigated biomarkers as compared to the individual markers, underlining the importance of investigating multiple markers simultaneously.

## Introduction

New prognostic biomarkers are warranted in colorectal cancer that could improve decisions for treatment of individual patients in addition to the current TNM (American Joint Committee on Cancer, AJCC [1]) staging system, as even patients with the same TNM classification present with large differences in patient survival and tumor recurrence [2], [3]. Epigenetic mechanisms have been identified as factors frequently deregulated in tumors and are attractive targets for biomarker research, because of their roles in regulating gene expression and their potentially reversible nature. Numerous changes in DNA methylation, histone modifications and their modifying enzymes have been described in various tumor types, including colorectal cancer [4]–[6]. In this study, we focused on expression of histone-modifying enzymes of the Polycomb-group (PcG) and their associated histone modification, trimethylation of lysine 27 on histone H3 (H3K27me3), in colorectal cancer tissues.

The PcG proteins act in large multi-protein complexes, the so-called Polycomb repressive complexes (PRC) 1 and 2 [7]. PcG proteins play an important role in embryonic development and cell proliferation [8], [9], and are also involved in inducing epithelial-mesenchymal transition (EMT) [10]. Aberrant expression of several PcG proteins and correlations with patient outcome have been reported in various cancers. For example, expression of BMI1 polycomb ring finger oncogene (BMI1), a component of PRC1 and an important factor in stem cells [11], [12], was found to be correlated to patient outcome in several types of cancer [13]–[16]. Enhancer of zeste homolog 2 (EZH2), a key protein in the PRC2 complex, was also found to have prognostic value in several types of cancer [17]–[20]. SUZ12 polycomb repressive complex 2 subunit (SUZ12), another key component of the PRC2 complex, was found to have tumor-promoting functions in several cancers, including colon cancer [21]–[23]. The associated histone modification H3K27me3 was found to be higher expressed in tumor tissues, and to be associated with better prognosis in non-small cell lung cancer [24] and breast cancer [19].

Using immunohistochemical staining (IHC) and semi-automated scoring, we studied the expression of PcG proteins EZH2, BMI1 and SUZ12 and their associated histone modification H3K27me3 in a cohort of 247 TNM stage I-III colorectal cancer patients, in correlation with clinical outcome. As the PcG proteins act together on the same histone modification, we hypothesized the combination of all four markers would be more informative with respect to clinical outcome as compared to each of the individual markers.

## Materials and Methods

### Patient selection

Tumor tissues were collected from a consecutive series of 408 colorectal cancer patients who underwent surgical resection of their primary tumor at the Leiden University Medical Center (LUMC) between 1991 and 2001. Patients who underwent preoperative treatment, who had bilateral tumors, or a history of cancer other than basal cell carcinoma or *in situ* tumors, were excluded from the study analyses. In addition, we included only patients with a histologically proven TNM stage I-III colorectal carcinoma, as determined by an experienced pathologist. This resulted in a study cohort of 259 patients, with a mean follow-up of 8.6 years. Clinicopathological data were available for all patients in the study cohort. Data were right-censored when patients were alive or free of recurrence at their last follow-up date. Patient characteristics are displayed in Table 1. Patient records information was anonymized and de-identified prior to analysis according to national ethical guidelines (“Code for Proper Secondary Use of Human Tissue”, Dutch Federation of Medical Scientific Societies), and approved by the Medical Ethical Committee of the Leiden University Medical Center (LUMC). This study was performed according to the REMARK guidelines (NCI-EORTC) [25].

**Table 1:** Patient characteristics of the study cohort.

		All patients		Study cohort		
		*(n = 408)*		*(n = 247)*		
		N	(%)	n	(%)	*P-values*
Age at randomization						
	<50	45	11.0	32	13.0	
	50–75	267	65.4	155	62.8	
	> = 75	96	23.6	60	24.3	0.69
Gender						
	Male	202	49.5	127	51.4	
	Female	206	50.5	120	48.6	0.66
TNM stage						
	I	78	19.0	52	21.1	
	II	149	36.7	110	44.5	
	III	114	27.9	85	34.4	0.21
	IV	67	16.4			
Tumor location						
	Colon	289	71.0	181	73.3	
	Rectum	119	29.0	66	26.7	0.60
Tumor size						
	Mean (cm)	4.68		4.71		
	Standard error	2.22		1.53		0.95
MSS status						
	MSS	275	67.2	169	68.4	
	MSI	46	11.2	34	13.8	
	Unknown	87	21.6	44	17.8	0.76
Tumor in follow up						
	No	347	85.0	209	84.6	
	Yes	61	15.0	38	15.4	0.91
Adjuvant therapy						
	No	323	79.2	199	80.6	
	Yes	85	20.8	48	19.4	0.97

Patient characteristics are shown for both the study cohort (n = 247) and the complete colorectal cancer series (n = 408). Patient selection was based on availability of FFPE tissues and available data for all four studied markers. The study cohort selection was representative for the entire colorectal cancer series. P-values represent the results of Student’s t-tests. For TNM stage, only tumor stage I-III of the complete patient cohort were compared to the patients in the study cohort.

### Tissue microarray construction and immunohistochemistry

Formalin-fixed paraffin-embedded (FFPE) tumor tissues from each of the patients in the consecutive series of colorectal cancer patients (n = 408) were collected from the LUMC pathology archives and used to construct a tissue microarray (TMA), as described previously [26]. Sections of 4 µm were cut from each TMA block and used for IHC staining. Histologically normal colorectal tissues, as determined by an experienced pathologist, from 47 patients with corresponding tumor tissues included in this study were also collected and prepared for IHC. The following antibodies were used for IHC: anti-EZH2 (612667, BD Biosciences, San Jose, CA, USA), anti-BMI1 (ab14389, Abcam, Cambridge, UK), anti-SUZ12 (ab12073, Abcam) and anti-H3K27me3 (ab6002, Abcam). All antibodies have validated for use in immunohistochemistry by Western blot [27]–[30]. All primary antibodies were used at predetermined optimal dilutions and IHC was performed using a standard IHC protocol [31]. Briefly, endogenous peroxidase was blocked by incubating the sections in a 0.3% solution of hydrogen peroxide (in PBS) for 20 min. Antigen retrieval was performed by heating the sections for 10 min at 95°C in a citrate buffer (pH 6; pH Low Target Retrieval Solution, Dako, Glostrup, Denmark) for EZH2, BMI-1 and H3K27me3 and by heating the sections for 10 min at 95°C in a Tris-EDTA buffer (pH 9; pH High Target Retrieval Solution, Dako) for SUZ12. TMA sections were incubated with the respective primary antibodies overnight (16 hrs). Staining was visualized using the Dako REAL EnVision Detection System, Peroxidase/DAB+, Rabbit/Mouse (Dako). The stained TMA sections were scanned using a 20x magnification on the semi-automated Ariol system (Leica Microsystems, Wetzlar, Germany). Tumor cell areas (tumor tissues) and colon epithelium (in normal tissues) were marked on the computer screen upon visual inspection, followed by careful training of the Ariol system to correctly identify positively stained and negative nuclei within the marked tissue areas, for each of the markers separately. Nuclear expression, defined as the percentage of positively stained nuclei in the marked area of each tissue core, was then assessed by the Ariol software. Several random cores were evaluated for each TMA section by visual inspection after automatic analysis in order to verify correct identification of positively stained nuclei.

### Statistical analyses

Data were analyzed in consultation with a statistician (H.P.) using SPSS 20.0 for Windows (SPSS Inc, Chicago, USA). 12 patients were excluded from the statistical analyses, as not all data of all four markers was available for these patients, resulting in a final patient cohort consisted of 247 patients. As the individual marker data were not normally distributed (Shapiro Wilk-test), non-parametric Wilcoxon signed-rank tests were performed to assess the differences in nuclear expression between tumor and paired normal tissues (n = 47) for each of the markers. Spearman’s signed rank correlation analyses were performed to investigate the correlation between nuclear expression of the individual PcG proteins and histone modification H3K27me3. Cox proportional hazard trend analyses were performed for univariate and multivariate survival analyses of individual markers. Covariates included in all multivariate analyses were age at operation, gender, TNM tumor stage (tumor stages I-III), tumor location, tumor size, microsatellite stability (MSS) status. Covariates “tumor in the follow up” and “adjuvant therapy” were entered as time-dependent covariates. Overall survival (OS) was defined as the time from surgery until death (by any cause). Disease-free survival (DFS) was defined as the time from surgery until the occurrence of a second primary colorectal tumor, locoregional recurrence or distant recurrence, or death by colorectal cancer. Locoregional recurrence-free survival (LRRFS) was defined as the time from surgery until the occurrence of a locoregional recurrence or death by cancer. Distant recurrence-free survival (DRFS) was defined as the time from surgery until the occurrence of a distant recurrence or death by cancer.

On the basis of the skewed distribution of expression data of each of the individual markers, the median expression was used to divide the patients into high expression (above-median) and low expression (below-median) groups. The four markers were then combined into a new variable, based on the number of markers showing high nuclear expression, resulting in the following grouping: all low (group 1), 1 high (group 2), 2 high (group 3), 3 high (group 4) and all high (group 5). Univariate and multivariate Cox proportional hazard analyses were performed using the group numbers as a categorical variable, using group 1 (all low) as the reference group. Based on these results we decided to combine patient groups 2, 3, and 4 into one patient group; all further statistical analyses were performed using three patient groups. In addition to the Cox proportional hazard analyses, trend analyses were performed using the group numbers as continuous variables to assess the influence of the combined markers on patient survival and tumor recurrence. Resulting hazard ratios (HR) represent the HR for each unit of increase (increase in group number). Cumulative incidence curves were made for DFS, LRRFS and DRFS, accounting for competing risks [32]. Kaplan-Meier curves were used to visualize differences between the three patient groups for OS. For all statistical analyses, two-sided p-values ≤0.05 were considered as statistically significant, and p-values 0.05<p≤0.1 were considered a trend.

## Results

### Expression in tumor versus paired normal colorectal tissues

**Figure 1:**
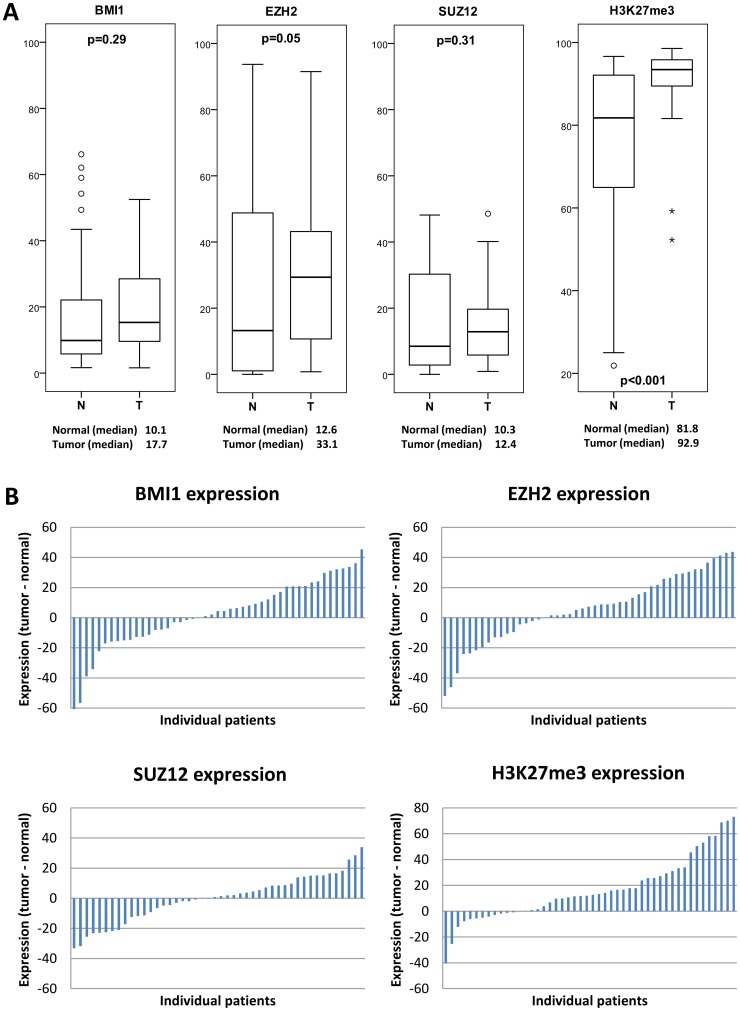
Differences in nuclear expression between normal and tumor tissues of individual markers. (A) Displayed are differences in nuclear expression, measured as the percentage of positively stained nuclei, between normal and tumor tissues (n = 47). Boxplots show the median and range of expression of each of the individual markers in normal (N) and tumor (T) samples. The median percentages of positive nuclei are given for each of the markers. P-values represent statistical differences between normal and tumor samples, calculated using the Wilcoxon signed rank test. (B) Histograms show the difference in expression between tumor and paired normal tissues (y-axis) for each of the individual patients (x-axis). Differences in expression were calculated as follows: expression difference  =  expression in tumor tissue – expression in normal tissue. Negative values indicate higher expression in normal tissues, positive values indicate higher expression in tumor tissues.

Nuclear expression of all individual markers (EZH2, BMI1, SUZ12 and H3K27me3) in tumor tissues was compared to nuclear expression in paired normal colorectal tissues. When analyzing expression differences in the study cohort as a whole, only median H3K27me3 and EZH2 expression were significantly different between tumor and normal tissues (p<0.001 and p = 0.05, respectively; Figure 1A). In individual tumors, however, all markers showed marked differences in expression compared to their normal counterparts (Figure 1B). Survival analyses based on below- or above-median expression in the normal tissues did not show differences in patient survival or tumor recurrence (data not shown).

### Individual marker analyses in tumor tissues

**Figure 2:**
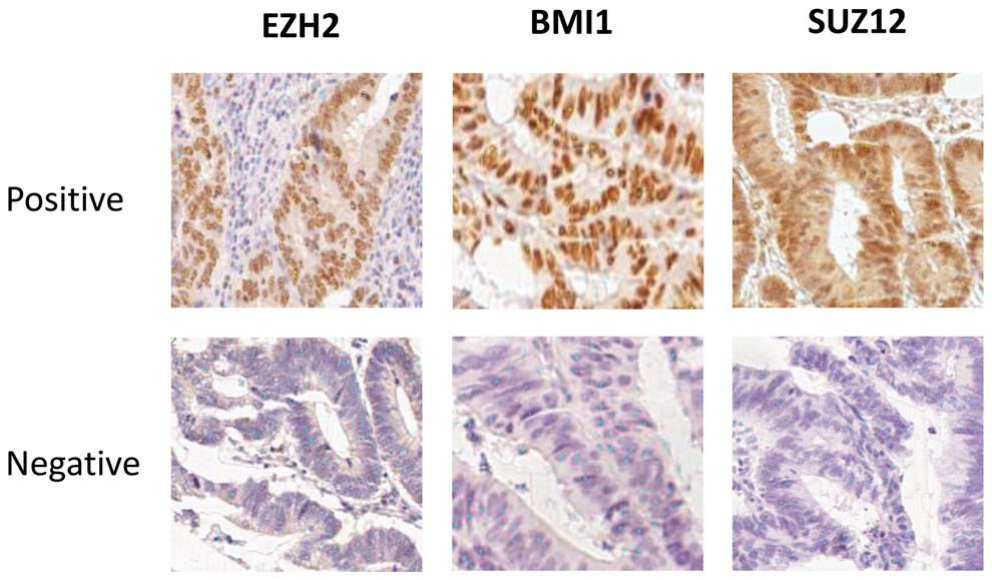
Identification of positive and negative tumor cell nuclei by the Ariol system. The Ariol system trainer overlay shows correct identification of positive (indicated by yellow dots) and negative (blue dots) nuclei in tumor cores. TMA slides were scanned using a 20x magnification. Shown for all markers are positively stained tumor cores (*top row*) and negative tumor cores (*bottom row*). The Ariol system was trained to identify positive and negative cells for each marker individually.

Examples of identification of positive and negative tumor cell nuclei for each of the individual markers by the Ariol system are shown in Figure 2. We first analyzed if the expression of histone modification H3K27me3 was correlated to the expression of the individual PcG proteins. The nuclear expression (percentage of positive nuclei) of H3K27me3 was indeed positively correlated with expression of EZH2 (p<0.001), BMI1 (p<0.001) and SUZ12 (p = 0.05). No correlation was observed between the expression of the individual markers and TNM tumor stage. For survival analyses, patients were divided into low and high expression groups based on the median expression of each of the individual markers, as given in Figure 1A. In survival analyses of individual markers, BMI1 showed strong correlations to patient survival (OS and DFS) and tumor recurrence (LRRFS and DRFS) in both univariate and multivariate analyses (Table 2). EZH2 and H3K27me3 showed significant correlations for DFS only. For all three markers (BMI1, EZH2 and H3K27me3), high expression was associated with better patient survival as compared to the patients showing low expression, with p-values for DFS of p = 0.07 (BMI1), p = 0.04 (EZH2) and p = 0.06 (H3K27me3). SUZ12 did not show any differences in patient survival or tumor recurrence based on low or high expression of the marker in tumor tissues.

**Table 2:** Univariate and multivariate survival analyses individual markers.

			EZH2	BMI1	SUZ12	H3K27me3
OS	Univariate	p-value	*0.07*	**0.05**	0.9	0.5
		HR	0.74	0.73	1.03	0.89
		(95% CI)	(0.54–1.03)	(0.53–1.00)	(0.73–1.47)	(0.64–1.24)
	Multivariate	p-value	0.3	**0.009**	0.3	0.5
		HR	0.84	0.62	0.83	0.88
		(95% CI)	(0.60–1.18)	(0.44–0.89)	(0.57–1.20)	(0.62–1.24)
DFS	Univariate	p-value	**0.04**	*0.08*	0.8	*0.07*
		HR	0.64	0.68	0.95	0.66
		(95% CI)	(0.42–0.99)	(0.44–1.04)	(0.60–1.49)	(0.42–1.03)
	Multivariate	p-value	*0.08*	**0.03**	0.2	**0.05**
		HR	0.67	0.61	0.71	0.64
		(95% CI)	(0.43–1.05)	(0.39–0.96)	(0.44–1.17)	(0.41–0.99)
LRRFS	Univariate	p-value	*0.06*	**0.03**	0.8	0.2
		HR	0.67	0.63	1.06	0.76
		(95% CI)	(0.44–1.03)	(0.41–0.96)	(0.67–1.68)	(0.49–1.18)
	Multivariate	p-value	*0.1*	**0.005**	0.6	0.2
		HR	0.71	0.52	0.88	0.76
		(95% CI)	(0.46–1.11)	(0.33–0.82)	(0.55–1.42)	(0.49–1.19)
DRFS	Univariate	p-value	**0.04**	*0.06*	0.6	0.4
		HR	0.64	0.66	1.11	0.83
		(95% CI)	(0.42–0.99)	(0.48–1.02)	(0.69–1.77)	(0.53–1.29)
	Multivariate	p-value	*0.1*	**0.02**	0.7	0.6
		HR	0.73	0.58	0.91	0.89
		(95% CI)	(0.46–1.15)	(0.36–0.92)	(0.56–1.48)	(0.57–1.42)

Shown are the results of the univariate and multivariate analyses of all individual markers, with all p-values and hazard ratios (HR) and their 95% confidence intervals (95% CI). The "low expression" group was used as the reference group. Covariates included in all multivariate analyses were age at operation, gender, TNM tumor stage, tumor location, tumor size, microsatellite stability (MSS) status, tumor in the follow up and adjuvant therapy. OS  =  overall survival, DSS  =  disease-specific survival, LRRFS  =  locoregional recurrence-free survival, DRFS  =  distant recurrence- free survival. Significant p-values are indicated in **bold**, p-values showing a trend (0.05≤0.1) in *Italic*.

### Combined markers

We hypothesized that combining multiple markers would result in better stratification of patients. Therefore, we performed statistical analyses on combinations of the histone-modifying enzymes. These analyses showed that indeed combining multiple markers resulted in statistically significant differences between the patient groups and more pronounced hazard ratios, indicating a more pronounced effect on patient survival. The combination of histone-modifying enzymes EZH2 and BMI1 showed significant differences for both patient survival and recurrence-free survival, with p = 0.02 (HR = 0.72; 95% CI 0.54–0.94) for DFS and p = 0.012 (HR = 0.71; 95% CI 0.54–0.92) for LRRFS in multivariate analyses. Combining EZH2 and SUZ12 showed a trend for DFS in multivariate analyses, with p = 0.08 (HR = 0.77; 95% CI 0.57–1.04). The combination of BMI1 and SUZ12 showed significant differences for patient survival, with p = 0.02 (HR = 0.76; 95% CI 0.61–0.96) for overall survival and p = 0.05 (HR = 0.73; 95% CI 0.54–1.00).

**Figure 3:**
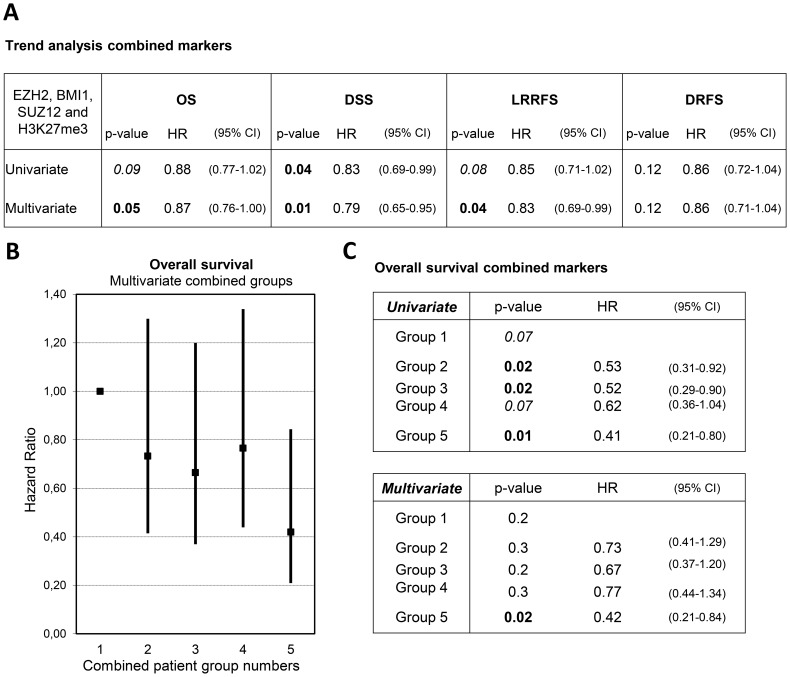
Trend analyses and hazard ratios of combined marker groups. Patient groups were made based on the number of markers showing high expression: all low (group 1), one high (group 2), two high (group 3), three high (group 4) and all high (group 5). (A) Univariate and multivariate Cox regression trend analyses were performed using the combined marker groups as continuous variables. Hazard ratios per unit increase of each of the patient groups were plotted (B) and listed (C) for both the univariate and multivariate Cox regression trend analyses using the combined marker groups as categorical variables. The numbers of patients in the individual patient groups were: group 1 (n = 28), group 2 (n = 59), group 3 (n = 55), group 4 (n = 74) and group 5 (n = 31).

**Figure 4:**
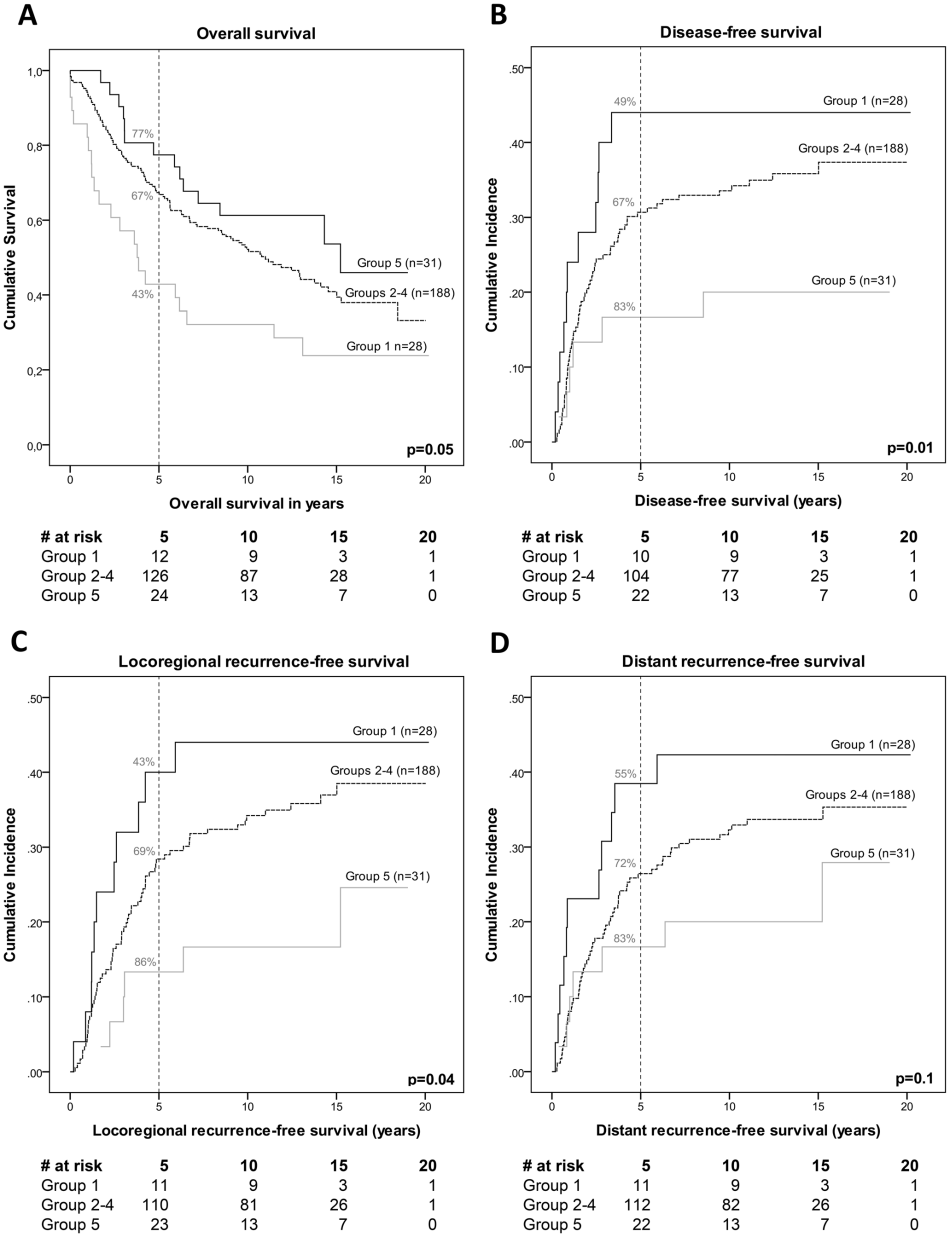
Kaplan-Meier and cumulative incidence curves of the combined markers. Combined marker (EZH2, BMI1, SUZ12 and H3K27me3) expression groups were divided into three patient groups: group 1, groups 2–4 and group 5. The numbers of patients in the individual patient groups were: group 1 (n = 28), group 2 (n = 59), group 3 (n = 55), group 4 (n = 74) and group 5 (n = 31). Kaplan-Meier curves were made for overall survival (A) and cumulative incidence curves are shown for disease-free survival (B), locoregional recurrence-free survival (C) and distant recurrence-free survival (D). 5-year survival rates are given for each patient group. Tables below the curves indicate the numbers at risk (#) per group for the different time points.

Because the three PcG proteins act together in multi-protein complexes to regulate H3K27me3 expression, we hypothesized that combining all markers (BMI1, EZH2, SUZ12 and H3K27me3) into one variable would result in even better stratification of patients. Patients were divided into five groups based on the number of markers showing high nuclear expression. This resulted in the following patient groups: all low (group 1), one high (group 2), two high (group 3), three high (group 4) and all high (group 5). Patient characteristics of the 5 patient groups were comparable to the study cohort (Table S1). Multivariate trend analyses of the combined markers, using the patient group numbers as continuous variables, showed overall hazard ratios of 0.79–0.88 for each additional marker showing high nuclear expression in both univariate and multivariate analyses, indicating better patient survival and lower chances of tumor recurrence for each additional marker showing high expression (Figure 3A). When the patient group numbers were entered as categorical variables, a similar trend was observed (Figures 3B and 3C). Generally, hazard ratios for OS decreased with increasing group number, indicating a better patient survival when more markers were highly expressed compared to the “all low” expression group (group 1). Patients showing high expression of all markers, the “all high” group (group 5), showed the best overall survival (p = 0.01, HR = 0.42, 95% CI 0.21–0.84) as compared to reference group 1, which showed the shortest survival. Groups 2, 3 and 4 showed similar hazard ratios (Figures 3B and 3C), which was also reflected in the Kaplan-Meier survival curves that run close together as compared to the survival curves of groups 1 and 5. Therefore, we decided to combine the three patient groups 2,3 and 4 into one group, resulting in three patient groups (group 1, groups 2–4 and group 5). Kaplan-Meier curves and cumulative incidence plots showed significant differences between the three resulting patient groups for OS, DFS, LRRFS and a trend for DRFS, which were also reflected in the 5-year survival rates (Figure 4). The best patient survival and longest recurrence-free periods were observed for patients showing high expression of all four markers (“all high”, group 5) in the tumor samples, with 5-year survival rates of 77% for OS, 83% for DFS and DRFS, and 86% for LRFS. Patients in combined groups 2–4 showed shorter OS, DFS and LRFS, with 5-year survival rates of 67% for OS and DFS, 69% for LRFS and 72% for DRFS. Patients in the “all low” group (group 1) showed significantly shorter OS, DFS and LRFS compared to either of the other patient groups, with 5-year survival rates of 43% for OS and LRFS, 49% for DFS and 55% for DRFS. Taken together, 5-year survival rates were lower when more markers showed low expression. The hazard ratios in both univariate and multivariate also reflected these findings (Table 3): group 5 shows the lowest hazard ratio as compared to reference group 1 (for example, multivariate HR = 0.23 (0.08–0.67) for DFS). This indicates a lower risk of an event (patient death or locoregional tumor recurrence) for patients in the “all high” group for OS, DFS and LRFS. For DRFS, statistically significant results were only observed in univariate analyses.

**Table 3:** Univariate and multivariate analyses combined markers.

		Univariate			Multivariate		
		p-value	HR	(95% CI)	p-value	HR	(95% CI)
	Group 1	**0.018**			**0.05**		
	Groups 2–4	**0.014**	0.56	(0.35–0.89)	0.21	0.72	(0.44–1.19)
OS	Group 5	**0.009**	0.41	(0.21–0.80)	**0.01**	0.42	(0.21–0.84)

		Univariate			Multivariate		
		p-value	HR	(95% CI)	p-value	HR	(95% CI)
	Group 1	*0.07*			**0.03**		
DFS	Groups 2–4	0.1	0.64	(0.33–1.21)	0.2	0.61	(0.31–1.22)
	Group 5	**0.02**	0.32	(0.12–0.86)	**0.007**	0.23	(0.08–0.67)

		Univariate			Multivariate		
		p-value	HR	(95% CI)	p-value	HR	(95% CI)
	Group 1	*0.06*			**0.03**		
LRRFS	Groups 2–4	0.2	0.64	(0.34–1.22)	0.96	1.02	(0.49–2.09)
	Group 5	**0.02**	0.31	(0.11–0.83)	**0.02**	0.30	(0.11–0.84)

		Univariate			Multivariate		
		p-value	HR	(95% CI)	p-value	HR	(95% CI)
	Group 1	0.12			0.24		
DRFS	Groups 2–4	0.11	0.59	(0.31–1.13)	0.66	0.85	(0.41–1.75)
	Group 5	**0.05**	0.38	(0.15–0.98)	0.11	0.44	(0.16–1.20)

Results of Cox proportional hazard univariate and multivariate analyses are shown for combined markers EZH2, BMI1, SUZ12 and H3K27me3, with p-values and hazard ratios with their 95% confidence intervals. Patient groups were made based on the number of markers showing high expression: all low (group 1), one, two or three high (groups 2–4) and all high (group 5). Group 1 was used as the reference group. Covariates included in all multivariate analyses were age at operation, gender, TNM tumor stage, tumor location, tumor size, microsatellite stability (MSS) status, tumor in the follow up and adjuvant therapy. Significant values are shown in **bold**, p-values showing a trend (between 0.5 and 1.0) in *Italic*.

## Discussion

In addition to gene mutations, aberrant expression patterns of epigenetic regulators have been recognized as crucial events in the tumorigenic process, resulting in marked changes in gene expression. Changes in the expression of these epigenetic regulators include DNA methyltransferases and consequent changes in DNA methylation profiles, and histone-modifying enzymes and resulting changes in their corresponding histone modifications. In this study, we investigated the expression of three PcG proteins (EZH2, BMI1 and SUZ12) and associated histone modification H3K27me3 in colorectal cancer tissues. Aberrant expression of each of these histone-modifying enzymes, and of histone modification H3K27me3, has been indicated to contribute to tumorigenesis in several types of cancer and has been correlated to patient outcome [11]–[24]. Studies in literature show conflicting results regarding the prognostic value of the Polycomb group proteins in colorectal cancer. For example, high EZH2 expression has been associated with poor prognosis in a series of colorectal cancer patients by Wang *et al*. [33], whereas high EZH2 expression was found to be associated with better relapse-free survival in colon cancer patients (but not in rectal cancer patients) by Fluge *et al*. [34]. In addition, high expression of BMI1 was found to correlate with good prognosis in breast cancer in a study by Pietersen *et al*. [35], whereas high BMI1 was associated with poor prognosis in colon cancer in a study by Du *et al*. [36]. In our study cohort, survival data for the individual markers showed that high expression of all markers was correlated with better patient survival and longer recurrence-free periods as compared to patients showing low expression. The results found in this study correspond to our previous findings that high expression of H3K27me3 was associated with better patient survival in rectal tumors [4]. In this study, we showed that high expression of H3K27me3 was indeed associated with better patient survival and longer recurrence-free periods. As we showed that the expression of the PcG proteins was directly related to the expression of H3K27me3, we expected a similar correlation of expression of the PcG proteins with clinical outcome, which was indeed confirmed by the results presented in this manuscript. High levels of H3K27me3, because of aberrant expression of PcG proteins, might prevent aberrant expression of oncogenes, activation of retrotransposon sequences (such as LINE-1; [4]), and result in other (epi)genomic events that promote tumor aggressiveness.

In addition to the individual markers, combinations of PcG proteins in correlation with patient outcome have been studied by several research groups. For example, co-expression of EZH2 and BMI1 was reported to be associated with poor prognosis in various cancers [37]–[39]. In contrast, overexpression of EZH2 and BMI1 were reported to have different influences on patient prognosis in breast cancer [35], and was found to have no prognostic value in urothelial carcinoma of the bladder [40]. In our colorectal cancer study cohort, all combinations of histone-modifying enzymes showed prognostic value. In order to obtain more information about epigenetic pathways with potential prognostic value in colorectal cancer, we performed multivariate survival analyses using combined expression data of multiple PcG proteins (EZH2, BMI1 and SUZ12) and their associated histone modification H3K27me3. Combining the three PcG proteins and their associated histone modification resulted in significantly better stratification of patient groups as compared to the individual markers. In combined marker analyses, the best patient survival and longest recurrence-free periods were observed for patients showing high expression of all four markers (“all high”) in the tumor samples. Patients in the “all low” group showed significantly shorter OS, DFS and LRFS compared to either of the other patient groups. The results of the combined marker analyses underline the co-operation of these three enzymes in PcG complexes, and thus provide a better risk stratification of patients.

In addition to the roles of EZH2, BMI1 and SUZ12 in epigenetic regulation of chromatin structure and gene expression, direct regulation of protein function has been described for EZH2 and BMI1, including protein phosphorylation and ubiquitination. A cytosolic EZH2 and SUZ12-containing methyltransferase complex has been linked to actin polymerization, an important process in cell proliferation [41]. Shuttling of the EZH2 and SUZ12 containing complex between different cellular compartments may explain the weak cytosolic staining observed for EZH2 and SUZ12 in addition to the strong nuclear staining for these markers, as compared to the strict nuclear staining observed for BMI1. Another non-histone protein methylated by EZH2 is cardiac transcription factor GATA4. Methylation reduces its transcriptional activity, resulting in inhibition of proper cardiac development [42]. These examples indicate that aberrant expression of these PcG proteins influences key processes such as gene transcription and cell proliferation, promoting the transformation of normal cells into tumor cells.

In conclusion, we showed that combined expression of PcG proteins EZH2, BMI1 and SUZ12 and their associated histone modification H3K27me3 has prognostic value in our colorectal cancer study cohort. Combined marker expression resulted in better stratification of patients as compared to the individual markers and hence provides more insight into the roles of these epigenetic proteins and –modifications in colorectal cancer. Other combinations of epigenetic mechanisms should be investigated in colorectal cancer to further unravel the underlying biology in individual tumors. This will advance the search for new biomarkers to be used in a clinical setting in order to better classify patients for treatment.

## Supporting Information

Table S1 **Patient characteristics of all patient groups used in combined-marker analyses.** Patient characteristics are shown for all patient groups as used in the combined-marker analyses. The patient groups show comparable patient characteristics to the complete study cohort of 247 patients (Table 1). P-values represent the Jonckheere-Terpstra test used to test if samples came from the same distribution. For the variable "tumor size", a one-way ANOVA test was performed to test for statistical differences between the patient groups.
(DOC)
