# Supplementary material for: Prognostic Value of Polycomb Proteins EZH2, BMI1 and SUZ12 and Histone Modification H3K27me3 in Colorectal Cancer
Source: PLoS One. 2014 Sep 22;9(9):e108265. doi: 10.1371/journal.pone.0108265 (PMC4171510; doi:10.1371/journal.pone.0108265)
Supplement: Table S1 — Patient characteristics of all patient groups used in combined-marker analyses. Patient characteristics are shown for all patient groups as used in the combined-marker analyses. The patient groups show comparable patient characteristics to the complete study cohort of 247 patients (Table 1). P-values represent the Jonckheere-Terpstra test used to test if samples came from the same distribution. For the variable "tumor size", a one-way ANOVA test was performed to test for statistical differences between the patient groups. (DOC) [file pone.0108265.s001.doc]

| **Supplementary Table 1** Patient characteristics of all patient groups used in combined-marker analyses | | | | | | | | | |  |  |  |
| --- | --- | --- | --- | --- | --- | --- | --- | --- | --- | --- | --- | --- |
|  |  |  |  |  |  |  |  |  |  |  |  |  |
|  | | ***Group 1*** | | ***Group 2*** | | ***Group 3*** | | ***Group 4*** | | ***Group 5*** | |  |
|  |  | *(n=28)* | | *(n=59)* | | *(n=55)* | | *(n=74)* | | *(n=31)* | |  |
|  | | **N** | **(%)** | **N** | **(%)** | **N** | **(%)** | **N** | **(%)** | **N** | **(%)** | ***P-values*** |
| Age at randomization | |  | |  | |  | |  | |  | |  |
|  | <50 | 1 | 3.6 | 9 | 15.3 | 9 | 16.4 | 10 | 13.5 | 3 | 9.7 |  |
|  | 50-75 | 15 | 53.6 | 41 | 69.5 | 36 | 65.5 | 47 | 63.5 | 16 | 51.6 |  |
|  | >=75 | 12 | 42.9 | 9 | 15.3 | 10 | 18.2 | 17 | 23.0 | 12 | 38.7 | 0.81 |
| Gender | |  | |  | |  | |  | |  | |  |
|  | Male | 14 | 50 | 28 | 47.5 | 27 | 49.1 | 36 | 48.6 | 15 | 48.4 |  |
|  | Female | 14 | 50 | 31 | 52.5 | 28 | 50.9 | 38 | 51.4 | 16 | 51.6 | 0.99 |
| TNM stage | |  | |  | |  | |  | |  | |  |
|  | I | 7 | 25 | 12 | 20.3 | 12 | 21.8 | 14 | 18.9 | 7 | 22.6 |  |
|  | II | 9 | 32.1 | 27 | 45.8 | 25 | 45.5 | 31 | 41.9 | 18 | 58.1 |  |
|  | III | 12 | 42.9 | 20 | 33.9 | 18 | 32.7 | 29 | 39.2 | 6 | 19.4 | 0.56 |
| Tumor location | |  | |  | |  | |  | |  | |  |
|  | Colon | 18 | 64.3 | 44 | 74.6 | 40 | 72.7 | 61 | 82.4 | 18 | 58.1 |  |
|  | Rectum | 10 | 35.7 | 15 | 25.4 | 15 | 27.3 | 13 | 17.6 | 13 | 41.9 | 0.85 |
| Tumor size | |  | |  | |  | |  | |  | |  |
|  | Mean (cm) | 4.55 | | 4.50 | | 4.72 | | 4.89 | | 4.77 | |  |
|  | Standard error | 0.55 | | 0.31 | | 0.32 | | 0.37 | | 0.41 | | 0.91 |
| MSS status | |  | |  | |  | |  | |  | |  |
|  | MSS | 18 | 64.2 | 44 | 74.6 | 35 | 63.6 | 51 | 68.9 | 21 | 67.7 |  |
|  | MSI | 5 | 17.9 | 7 | 11.9 | 10 | 18.2 | 8 | 10.8 | 4 | 12.9 |  |
|  | Unknown | 5 | 17.9 | 8 | 13.6 | 10 | 18.2 | 15 | 20.3 | 6 | 19.4 | 0.73 |
| Tumor in follow up | |  | |  | |  | |  | |  | |  |
|  | No | 25 | 89.3 | 52 | 88.1 | 45 | 81.8 | 65 | 87.8 | 22 | 71 |  |
|  | Yes | 3 | 10.7 | 7 | 11.9 | 10 | 18.2 | 9 | 12.2 | 9 | 29 | 0.52 |
| Adjuvant therapy | |  | |  | |  | |  | |  | |  |
|  | No | 25 | 89.3 | 45 | 76.3 | 48 | 87.3 | 55 | 74.3 | 26 | 83.9 |  |
|  | Yes | 3 | 10.7 | 14 | 23.7 | 7 | 12.7 | 19 | 25.7 | 5 | 16.1 | 0.13 |
| Patient characteristics are shown for all patient groups as used in the combined-marker analyses. The patient groups show comparable patient characteristics to the complete study cohort of 247 patients (Table 1). P-values represent the Jonckheere-Terpstra test used to test if samples came from the same distribution. For the variable "tumor size", a one-way ANOVA test was performed to test for statistical differences between the patient groups. | | | | | | | | | | | | |
|  |  |  |  |  |  |  |  |  |  |  |  |  |
|  |  |  |  |  |  |  |  |  |  |  |  |  |
